# The value of signal intensity on T1-weighted chemical shift magnetic resonance imaging combined with proton magnetic resonance spectroscopy for the diagnosis of adrenal adenomas

**DOI:** 10.1590/0100-3984.2019.0095

**Published:** 2020

**Authors:** Claudio Carvalho Dalavia, Suzan Menasce Goldman, Homero José de Farias e Melo, Claudio Elias Kater, Jacob Szejnfeld, Wagner Iared, Sergio Aron Ajzen

**Affiliations:** 1 Escola Paulista de Medicina da Universidade Federal de São Paulo (EPM-Unifesp), São Paulo, SP, Brazil.

**Keywords:** Adrenal glands, Adrenal gland neoplasms, Adenoma/diagnostic imaging, Magnetic resonance imaging/methods, Proton magnetic resonance spectroscopy/methods, Glândulas suprarrenais, Neoplasias das glândulas suprarrenais, Adenoma/diagnóstico por imagem, Ressonância magnética, Espectroscopia de prótons por ressonância magnética

## Abstract

**Objective:**

To investigate the advantages of using modified signal intensity measurements on chemical shift imaging alone or in conjunction with proton magnetic resonance spectroscopy in the differential diagnosis of adrenal adenomas.

**Materials and Methods:**

This was a prospective study involving 97 patients with adrenal nodules or masses. The signal intensity index (SII) was calculated as [(*signal intensity on the in-phase image − signal intensity on the out-of-phase image)* ∕ (*signal intensity on the in-phase image*)] × *100%*. We determined the averages of the minimum, mean, and maximum signal intensity values measured on three consecutive images. When that was not possible (for smaller lesions), we used one or two images. We employed a region of interest that covered one half to two thirds of the mass. All indices were compared with metabolite ratios derived from spectroscopy: lactate/creatine; glutamine-glutamate/creatine; choline/creatine; choline/lipid; 4.0-4.3 ppm/Cr; and lipid/creatine.

**Results:**

Of the 97 patients evaluated, 69 were diagnosed with adenomas and 28 were diagnosed with nonadenomas. All SII measurements and spectroscopy-derived metabolite ratios were significant to the differentiation between adenomas and nonadenomas, except for the lipid/creatine and choline/lipid ratios. In 37.8% of the cases, it was not possible to perform spectroscopy. When it was possible, the lactate/creatine ratio was found to have higher accuracy than did the SII.

**Conclusion:**

Determining the SII and metabolite ratios increased the accuracy of the differential diagnosis of adrenal adenomas.

## Abstract (pt)

**Objetivo:**

Investigar as vantagens do uso de medições do índice de intensidade de sinal modificadas em imagens de deslocamento químico (chemical shift), isoladamente ou em conjunto com a espectroscopia por ressonância magnética de prótons, no diagnóstico diferencial de adenomas adrenais.

**Materiais e Métodos:**

Estudo prospectivo envolvendo 97 pacientes com nódulos ou massas adrenais. O índice de intensidade do sinal (SII) foi calculado como [(intensidade do sinal na imagem em fase – intensidade do sinal na imagem fora de fase) ∕ (intensidade do sinal na imagem em fase)] × 100%. Determinamos as médias dos valores mínimo, médio e máximo da intensidade do sinal medida em três imagens consecutivas. Quando isso não foi possível (para lesões menores), usamos uma ou duas imagens. Nós empregamos uma região de interesse que cobria de metade a dois terços da massa. Todos os índices foram comparados com razões metabólicas derivadas da espectroscopia: lactato/creatina, glutamato-glutamina/creatina, colina/creatina, colina/lipídio, 4,0–4,3 ppm/creatina e lipídio/creatina.

**Resultados:**

Dos 97 pacientes avaliados, 69 foram diagnosticados como adenomas e 28 foram diagnosticados como não adenomas. Todas as medições SII e razões de metabólitos derivados da espectroscopia foram significativas para a diferenciação entre adenomas e não adenomas, exceto as razões lipídio/creatina e colina/lipídio. Em 37,8% dos casos não foi possível realizar espectroscopia. Quando possível, a razão lactato/creatina apresentou maior precisão do que o SII.

**Conclusão:**

A determinação das razões SII e metabólitos aumentaram a acurácia do diagnóstico diferencial de adenomas adrenais.

## INTRODUCTION

Greater availability of imaging technology has led to a substantial increase in the rate of incidental detection of adrenal lesions in recent decades, ranging from 5% to 10%-rates similar to those reported in most autopsy studies^(1-5)^. Although most incidentalomas are benign, the adrenal gland is the fourth most common site of metastasis and it is therefore important to characterize adrenal masses accurately, which can have an impact on the staging and therapeutic management of the primary tumor^(6)^.

Chemical shift magnetic resonance imaging (MRI) is currently the most sensitive noninvasive method to differentiate adrenal adenomas from metastases, and it is often used when computed tomography (CT) findings are classified as indeterminate or preferably as a diagnostic tool^(7-9)^. In this method, the difference between the signal intensity of water protons and that of lipid protons is used in order to identify adenomas-which usually contain large amounts of intracellular lipid, whereas metastases do not. The signal intensity is additive on in-phase images and subtractive on out-of-phase images. Because of this difference, a voxel is “discarded” from the out-ofphase sequences and the magnitude of that drop is proportional to the amount of lipid in the tissue. Therefore, a nodule showing a significant out-of-phase signal intensity drop can be characterized as an adrenal adenoma^(8-12)^. The currently accepted signal intensity drop values for the characterization of adrenal adenomas range from 16.5%^(13-15)^ to 20.0%^(8-12)^, with a sensitivity of 71-87% and a specificity of 92-100%^(8-10,13-16)^.

Chemical shift MRI involves the placement of a region of interest (ROI) over the adrenal gland, covering one half to two thirds of the nodule surface area^(10,17,18)^. The mean number of pixels found in the measured area is calculated by specific software, and, on the basis of the values acquired from in-phase and out-of-phase sequences, the signal intensity index (SII) is calculated. On the same MRI scan, when the ROI is placed over the adrenal mass, the software also provides the average minimum and maximum pixel values in the ROI. However, a search of the imaging literature revealed no documentation of the routine use of these values in any calculation of the SII.

Proton magnetic resonance spectroscopy (^1^H-MRS) is a functional technique used in order to measure the metabolic activity of the lesions. With improvements in the technique, ^1^H-MRS can now be used in order to measure adrenal masses that are as small as 1.0 cm in diameter, as opposed to the larger (> 2.0 cm) masses evaluated in previous studies^(19,20)^. Although ^1^H-MRS is a promising method, respiratory movements still limit its application.

With the current methods, a small but important number of cases will remain indeterminate, and a percutaneous biopsy-considered the gold standard-may be indicated. However, biopsies are invasive methods, with documented risks of complications and an accuracy of 80-90%^(3)^.

The need to try to increase the accuracy of noninvasive diagnostic techniques, particularly in the diagnosis of adenomas, together with the possibility of measuring the signal drop in chemical shift MRI, led to the following guiding questions: Among the minimum, mean, and maximum values, which signal drop rate points to better visualization?; and, Does the use of chemical shift MRI in conjunction with ^1^H-MRS increase diagnostic accuracy in the differential diagnosis of adrenal nodules?

## MATERIALS AND METHODS

Between January 2004 and May 2012, 103 patients with adrenal nodules or masses were studied prospectively with chemical shift MRI and ^1^H-MRS. The study was approved by the local institutional review board (Reference no. 1639/11), and all participating patients gave written informed consent.

Patients were considered eligible if they had an adrenal nodule or mass ≥ 1.0 cm, had been previously evaluated with a dedicated adrenal CT or MRI protocol, had received histopathological confirmation by biopsy or surgery (in cases of pheochromocytoma, carcinoma, functioning adenomas, or uncharacteristic lesions), and, in cases diagnosed as adenoma, had CT- or MRI-confirmed lesion stability for more than 12 months.

For the analysis of the SII, we applied the following exclusion criteria: corrupted or incomplete data, which would preclude an appropriate analysis, coming from the workstation; and the presence of an exclusively cystic lesion (simple cyst). For the spectroscopic analysis, the exclusion criteria were failure to position the grid, a low signal-to-noise ratio, and non-coincident voxels.

### MRI and ^1^H-MRS

All MRI scans were acquired in a 1.5-T scanner (Magnetom Sonata; Siemens Medical Systems, Erlangen, Germany) with a phased-array body coil. No respiratory monitoring was used. We acquired T2-weighted sequences, together with chemical shift in-phase and out-of-phase T1weighted sequences, at the level of the adrenal mass. The T2-weighted images were obtained with a half-Fourier acquisition single-shot turbo spin-echo (HASTE) sequence. The physical parameters used are summarized in Table 1. The HASTE sequences were obtained in three orthogonal planes (axial, coronal, and sagittal) for three-dimensional mass localization and ^1^H-MRS planning. To determine the correct insertion of the volume of interest, three localization sagittal HASTE sequences were acquired, using the same physical parameters: at maximum inspiration, at maximum expiration, and during free breathing.

**Table 1:** Physical parameters used in order to acquire MRI sequences in a 1.5-T scanner.

Sequence	Number of images	Thickness (mm)	TR (ms)	TE (ms)	Matrix	FOV (mm)
T2-weighted						
Axial HASTE	24	3.0	1000-2000	87	167 × 256	280-350
Axial fat saturation HASTE	24	3.0	1000-2000	87	167 × 256	280-350
Coronal HASTE	20	3.0	1000-2000	82	167 × 256	280-350
Sagittal H.ASTE	13	3.0	1000-2000	87	167 × 256	280-350
Tl-weighted						
Axial in-phase chemical shift	24	3.0	173	4.8	167 × 256	280-350
Axial out-of-phase chemical shift	24	3.0	173	2.4	167 × 256	280-350
Axial gadolinium-enhanced VIBE	70	1.0	5.45	2.58	179 × 256	280-350

FOV, field of view; VIBE, volumetric interpolated breath-hold examination.

The ^1^H-MRS was performed using the T2-weighted HASTE images. Possible volume artifacts around the adrenal nodules or masses were minimized by performing ^1^H-MRS with multivoxel acquisition with a point-resolved spatially localized spectroscopy sequence provided by the manufacturer (PRESS-CSI; Siemens Medical Systems) to select the spectroscopic volume in the ROI. Initially, only the sagittal images acquired at maximum inspiration, at maximum expiration, and during free breathing were used. The multivoxel volume-of-interest grid was carefully positioned in the center of the lesion, with the use of all three sagittal sequences, to include as much of the lesion area as possible or, preferably, to include the entire lesion and part of adjacent adipose tissue. Subsequently, all images obtained in the three orthogonal planes (axial, coronal, and sagittal) at maximum expiration were used. We performed ^1^H-MRS of the adrenal gland with a voxel size of 0.56-3.38 cm^3^, a field of view of 100-150 cm, an echo time (TE) of 135 ms, a repetition time (TR) of 1500 ms, a delta factor of −3.4, and an acquisition time of 7-11 min. Six saturation region bands, with a thickness of 30 mm each, were positioned around the adrenal gland to minimize magnetic field inhomogeneity due to susceptibility effects from air in the lung parenchyma, in bony structures, in perirenal fat, and in fluids present in the biliary tree or kidneys. Spectral editing and water suppression was applied using water-selective pulses by frequency-selective coherence generation, known as the Mescher-Garwood approach^(21)^, so that the lipids present in the gland were present during the acquisition of ^1^H-MRS images. The total examination time, including patient positioning, acquisition of MR images, and spectroscopic data acquisition, was approximately 30 min.

### Analysis of signal intensity drops

All values required to calculate the SIIs were obtained by the same observer using a commercially available workstation (Leonardo; Siemens Medical Systems). For each patient, signal intensity measurements were obtained from the ROI over the adrenal mass on chemical shift inphase and out-of-phase T1-weighted sequences. Cystic, calcified, and necrotic components of the adrenal mass were excluded from the ROI to avoid deviations in the values obtained^(22)^.

**Figure 1:**
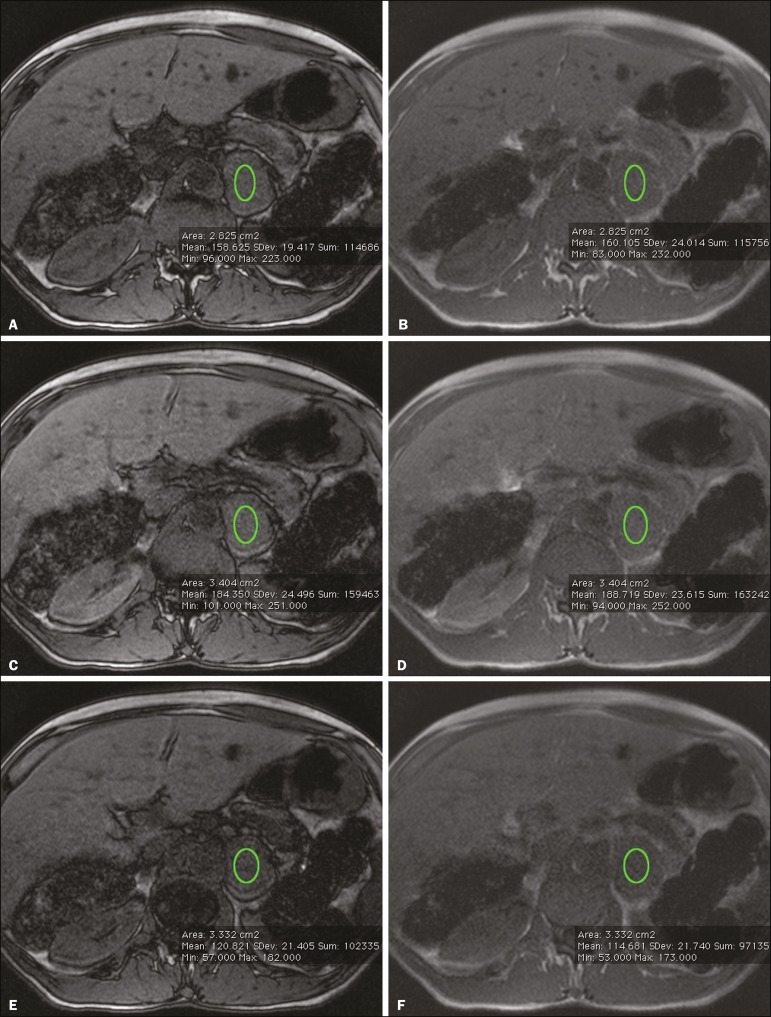
Chemical shift MRI. Placement of the ROI to cover one half to two thirds of the adrenal mass: axial out-of-phase (**A,C,E**) and in-phase (**B,D,F**) sequences.

First, the ROI was positioned to cover approximately one half to two thirds of the nodule surface area and fixed at a central position for in-phase and out-of-phase sequences. For each sequence, signal intensity was measured in three consecutive (axial) slices; minimum, mean, and maximum values were recorded (Figure 1). The minimum, mean, and maximum SII values were then calculated based on the average of the three minimum SII values (SII-minimum), mean SII values (SII-mean), and maximum SII values (SIImaximum), respectively. The following formula was used in order to calculate the SII:

$$\mathrm{SII}=\left[\left({\mathrm{SI}}_{\mathrm{in}-\mathrm{phase}}-{\mathrm{SI}}_{\mathrm{out}-\mathrm{of}-\mathrm{phase}}\right)/\left({\mathrm{SI}}_{\mathrm{in}-\mathrm{phase}}\right)\right]\times 100$$

where SI_in-phase_ represents the average of three values on in-phase sequences and SI_out-of-phase_ represents the average of three values on out-of-phase sequences. Thus, we determined the percentage signal intensity drop corresponding to each average obtained from the minimum, mean, and maximum values. Measurements were then made using a single (axial) slice for in-phase and out-of-phase sequences (Figure 1). The minimum, mean, and maximum values were recorded, after which we calculated the SII values, using the same formula shown above.

To better understand this population, we also divided the nodules or masses according to their size (diameter). For the SII analysis, we evaluated three size ranges: ≤ 2.0 cm; 2.1-4.0 cm; and > 4.0 cm.

### ^1^H-MRS data analysis

The graphs resulting from the ^1^H-MRS were analyzed by two observers in consensus. Data were processed using a dedicated ^1^H-MRS protocol on a workstation (Leonardo; Siemens Medical Systems) with a 1000-Hz Gaussian line-broadening filter. Fourier transformation in two spatial dimensions was performed using a Hanning filter. The ^1^H-MRS matrix was adjusted in all three orthogonal planes, and images were evaluated to determine which voxels were eligible for analysis. Individual voxels were considered eligible if 100% of their area was inside the tumor tissue. Voxels located in adjacent adipose tissue were not included in the spectral analysis. The spectral analysis was performed in the craniocaudal direction and from left to right.

**Figure 2:**
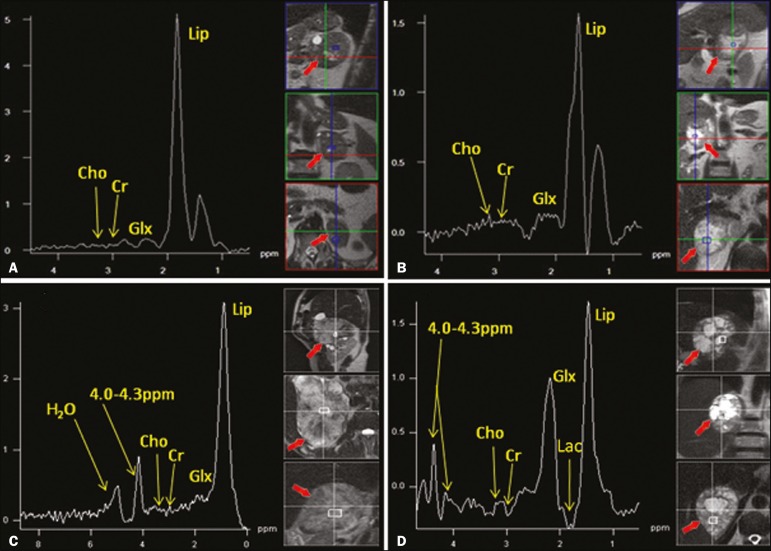
Spectroscopy graphs of four adrenal masses: adenoma (**A**), metastasis (**B**), pheochromocytoma (**C**), and carcinoma (**D**). The peaks of Cho, Cr, Lip, Glx, Lac, and H2O are shown in their respective curves. Red arrows indicate adrenal nodules in the different axes. Adapted from Melo et al.^(23)^.

After voxel selection, the x-axis was expanded to obtain a sufficient range (0.5-8.5 ppm) to correct the water peak with the other metabolites. The range was then reduced (to 0.5-4.7 ppm) in order to increase the spectral resolution. In both cases, ^1^H-MRS images were interpreted by visual inspection and amplitude peaks were measured for each metabolite of interest (Figure 2): lipid (Lip), 0.5-1.5 ppm; choline (Cho), 3.2 ppm; creatine (Cr), 3.03 ppm; catecholamines, 4.0-4.3 ppm; lactate (Lac), 1.33 ppm-out-of-phase, TE = 135 ms; and glutamine-glutamate (Glx), 2.1-2.5 ppm. After all amplitudes had been determined for each metabolite of interest, the following ratios were calculated: Lac/Cr; Glx/Cr; Cho/Cr; Cho/Lip; 4.0-4.3 ppm/Cr; and Lip/Cr.

### Statistical analysis

Statistical analyses were performed with the IBM SPSS Statistics software package, version 20.0 (IBM Corp., Armonk, NY, USA). For all tests, the level of significance was set at *p* < 0.05. Categorical variables are expressed as absolute and relative frequencies, whereas numerical variables are expressed as mean ± standard deviation or as median (range). Receiver operating characteristic curves were generated to determine the cutoff values of each parameter for differentiating adenomas from nonadenomas. Sensitivity, specificity, positive predictive value, negative predictive value, and accuracy were calculated on the basis of those cutoff values. A two-proportion Z-test was used in order to assess whether there were differences between the evaluation of the metabolite ratios (spectroscopy) and the evaluation of the SII values (chemical shift MRI). An analysis of sensitivity and specificity, including positive and negative predictive values, was then performed to determine whether a combination of the two methods would improve the differentiation between adenomas and nonadenomas. That analysis was performed with Stata software, version 13.1 (Stata Corp., College Station, TX, USA).

## RESULTS

**Figure 3:**
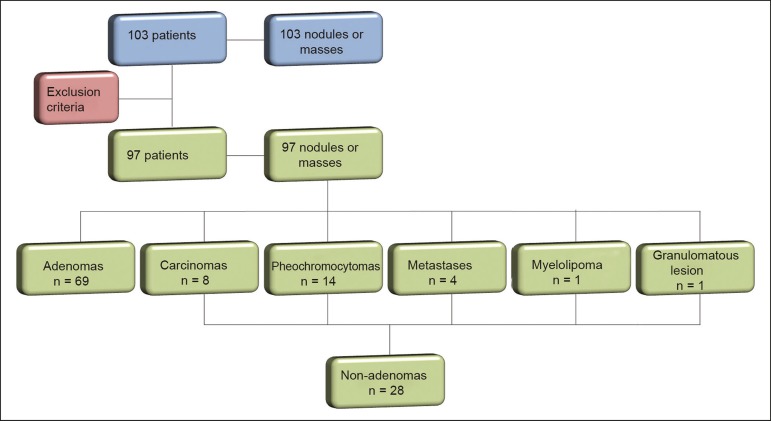
Study flow diagram.

Of the 103 patients, 5 were excluded because the data were corrupted or incomplete and 1 was excluded because of being diagnosed with a cystic lesion. Therefore, the final sample comprised 97 patients (27 men and 70 women), ranging in age from 13 to 87 years (mean, 57.51 ± 13.25 years). Among the 97 masses or nodules, diameters ranged from 0.97 cm to 11.48 cm (mean, 3.45 ± 2.45 cm). As can be seen in Figure 3, there were 69 adenomas and 28 nonadenomas, the latter group including eight carcinomas, 14 pheochromocytomas, four metastases, one myelolipoma, and one granulomatous lesion (histoplasmosis). Therefore, the prevalence of nonadenomas was 28.9%.

In eight nodules, it was not possible to acquire three consecutive slices because of the small size of the nodules. In those cases, we acquired two slices in four nodules (three adenomas and one pheochromocytoma) and one slice in three (all adenomas).

We excluded 36 lesions from the spectral analysis (25 adenomas, 5 pheochromocytomas, 3 metastases, 2 carcinomas, and 1 myelolipoma), because of grid positioning failure, a low signal-to-noise ratio, and non-coincident voxels. We included ganglioneuromas in the same group with pheochromocytomas, because the two are similar in terms of their embryological origin, imaging aspects, and spectroscopic behavior.

The mean SII values and metabolite ratios for the adenoma and nonadenoma groups are shown in Table 2. The cutoff values for each SII and metabolite ratio used for differentiating adenomas from nonadenomas are shown in Table 3. All SII values were statistically significant, with sensitivities ranging from 88.4-91.3% and specificities ranging from 67.9-75.0%. The metabolite ratios obtained from ^1^H-MRS were statistically significant, with sensitivities of 88.2-94.1% and specificities of 65.9-100%, except for the Lip/Cr and Cho/Lip ratios, which showed weak agreement and were unable to differentiate between adenomas and nonadenomas (*p* > 0.0001). The parameters with the highest sensitivity and specificity for differentiating adenomas from nonadenomas were the SII-minimum (sensitivity of 91.3% and specificity of 75.0%); the SII-mean (sensitivity of 88.4% and specificity of 75.0%); the Glx/Cr ratio (sensitivity of 94.1% and specificity of 90.9%); and the Lac/Cr ratio (sensitivity of 82.4% and specificity of 100%).

**Table 2:** Results of the measurements of SII and metabolite ratios in adenomas and nonadenomas.

Variable	Adenomas (n = 69)				Nonadenomas (n = 28)		
	Mean ± standard deviation	Minimum	Maximum		Mean ± standard deviation	Minimum	Maximum
Sll							
Minimum	68.3 ± 28.2	-55.4	96.9		15.4 ± 41.4	-63.5	86.2
Mean	54.3 ± 26.7	-34.5	100.0		15.7 ± 26.9	-16.2	70.4
Maximum	41.9 ± 26.7	-48.1	100.0		14.5 ± 26.1	-11.5	100.0
Metabolite ratios							
Lac./Cr	2.14 ± 1.68	0.00	7.34		-6.05 ± 7.37	-21.90	2.74
Glx/Cr	-5.06 ± 7.55	-33.83	1.73		0.48 ± 4.12	-11.29	7.65
Cho/Cr	0.92 ± 0.36	0.00	1.50		1.43 ± 0.43	0.96	2.36
4.0-4.3 ppm/Cr	0.96 ± 0.79	0.00	3.50		5.26 ± 7.33	0.98	4.74
Lip/Cr	27.7 ± 22.701	2.56	101.06		51.80 ± 80.91	32.85	363.77
Cho/Lip	0.11 ± 0.14	0.00	0.67		0.20 ± 0.27	0.02	1.14

**Table 3:** Cutoff points of each SII value and metabolite ratio for differentiating adenomas from nonadenomas.

Variable	Cutoff point	Sensitivity (%)	Specificity (%)	Positive predictive value (%)	Negative predictive value (%)	Accuracy	*P*-value
Sll							
Minimum	38.3	91.3	75.0	90.0	77.8	86.6	< 0.0001
Mean	25.7	88.4	75.0	89.7	72.4	84.5	< 0.0001
Maximum	10.9	88.4	67.9	87.1	70.4	82.5	< 0.0001
Metabolite ratios							
Lac/Cr	-0.52	82.4	100	100	93.62	95.09	< 0.0001
Glx/Cr	0.17	94.1	90.9	80.01	97.55	91.79	< 0.0001
Cho/Cr	1.06	88.2	65.9	50.02	93.52	72.12	< 0.0001
4.0-4.3 ppm/Cr	1.60	88.2	86.4	71.51	94.98	86.90	< 0.0001
Lip/Cr	28.14	64.7	68.2	44.05	83.31	67.22	0.135
Cho/Lip	0.08	70.6	63.6	42.87	84.83	65.55	0.037

When we stratified the cases by nodule diameter, we observed that there were 28 adenomas and 7 nonadenomas in the ≤ 2.0 cm group; 32 adenomas and 5 nonadenomas in the 2.1-4.0 cm group; and 9 adenomas and 16 nonadenomas in the > 4.0 cm group. We also observed that the sensitivity and specificity of the SII analysis were dependent on nodule size, in opposite ways. The method presented high sensitivity and low specificity for small nodules (≤ 2.0 cm) and for medium-sized nodules (2.1-4.0 cm), whereas, for large nodules (> 4.0 cm), its sensitivity ranged from 55.6% to 66.7% and its specificity was > 80% (Table 4).

**Table 4:** Cutoff points of each SII value for the differentiation between adenomas and nonadenomas, by nodule size (diameter).

Nodule diameter	Number of cases	Cutoff point	Sensitivity	Specificity	Positive predictive value	Negative predictive value	Accuracy
≤ 2.0 cm							
Sll-minimum	35	38.3	96.4	42.9	87.1	75.0	85.7
Sll-mean	35	25.7	96.4	28.6	84.4	66.7	82.9
Sll-maximum	35	10.9	96.4	28.6	84.4	66.7	82.9
2.1-4.0 cm							
Sll-minimum	37	38.3	93.8	60.0	93.8	60.0	89.2
Sll-mean	37	25.7	90.6	80.0	96.7	57.1	89.2
Sll-maximum	37	10.9	93.7	60.0	93.7	60.0	89.2
> 4.0 cm							
Sll-minimum	25	38.3	66.7	81.3	66.7	81.3	76.0
Sll-mean	25	25.7	66.7	87.5	75.0	82.4	80.0
Sll-maximum	25	10.9	55.6	81.3	62.5	76.5	72.0

## DISCUSSION

Chemical shift MRI is known to be superior to unenhanced CT in characterizing lipid-poor adenomas^(13)^. However, this method is useful only in cases of adrenal masses with attenuation values of 10-30 Hounsfield units at unenhanced CT, with a sensitivity of 89% for the identification of adenoma^(1)^. Therefore, additional criteria are needed to define the key role of MRI in differentiating atypical cases. In the present study, we employed complementary morphological characterization with functional spectroscopic analysis to add diagnostic information to MRI.

The prevalence of adenomas in the present study (71.1%) was within the 50-80% range reported in the literature^(15)^. We used different ways to calculate the SII based on minimum and maximum pixel values in the ROI, which are generally not considered in the SII calculation. The modifications included using the averages of three minimum, mean, and maximum signal intensity values measured, if possible, on three consecutive images (SIIminimum, SII-mean, and SII-maximum). In addition, all SIIs were compared with spectroscopy metabolite ratios for each type of adrenal mass. Among the SII values, the SII-minimum and SII-mean had the highest sensitivity and specificity in differentiating adrenal adenomas from nonadenomas. Among the metabolite ratios, the Lac/Cr and Glx/Cr ratios stood out as being highly accurate in characterizing adenomas, and were superior to all SII values.

Our objective in stratifying the nodules by diameter was to try to observe a greater or lesser concentration of fat in the adenomas and nonadenomas of different sizes. With the segmentation of the SII analysis by nodule size, we observed a significant increase in the specificity for the differentiation between adenomas and nonadenomas in parallel with an increase in nodule size, independent of the minimum, medium, or maximum. The sensitivity of SII, which was greater than 90% for nodules in the ≤ 2.0 cm and 2.1-4.0 cm groups, was considerably lower (55.6-66.7%) for those in the > 4.0 cm group. The differences in sensitivity and specificity related to nodule size could be attributable to the nonadenomas, which have different characteristics. Because the proportion of nonadenomas was smaller in the ≤ 2.0 cm group (20%) and in the 2.1-4.0 cm group (13.5%) than in the > 4.0 cm group (36%), it may be that the more homogeneous behavior of adenomas contributed to better sensitivity in the lower size ranges, whereas sensitivity was lower and specificity was higher for larger nodules. There is a need for studies involving larger samples and pathological confirmation in order to confirm that relationship.

Despite the decrease in sensitivity in nodules with a diameter greater than 4.0 cm, the diagnosis by imaging has a major influence on decisions regarding the treatment, given that the behavior of an adrenal nodule in relation to the neighboring structures (morphology), such as compression and invasion, will inform the surgical planning; at many centers, the protocol calls for resection even in the case of adenomas because of the risk of bleeding and the higher incidence of malignancy in such cases^(24)^. However, there are authors who recommend the follow-up of lesions that are typically benign, are nonfunctioning, and do not meet the criteria for malignancy^(24)^.

Spectroscopy may prove to be an effective method. In the cases in which it was possible to use spectroscopy, the Lac/Cr, Glx/Cr, Cho/Cr, and 4.0-4.3 ppm/Cr metabolite ratios showed good sensitivity (82.4-94.1%) and specificity (65.9-100%) for the differentiation between adenomas and nonadenomas. Only the Lip/Cr and Cho/Lip ratios had no significant cutoff points.

In the present study, we emphasized the Lac/Cr and Glx/Cr metabolite ratios, the specificity, positive predictive value, and accuracy of which were superior to those of all of the calculated forms of the SII. In a previous study conducted by our group, those metabolite ratios were found to be promising markers of malignancy^(25)^.

Although the metabolic analysis by MRS provides new insights in the study of adrenal nodules, it is still not practical. In our opinion, a method that can be employed in less than two thirds of cases should not be proposed for inclusion in the routine, requiring further study and development. The factors limiting its use in the present study were small-diameter nodules and non-coincident voxels.

Among the challenges of using spectroscopy is the need for suitable MRI hardware and software. The analysis time and the software need to evolve in order to make the routine use of spectroscopy feasible. The size of the mass will determine the time required for the analysis, the average analysis time being 30 min for smaller masses (≤ 4.0 cm in diameter), compared with 60-120 min for larger masses, even when the reader is quite experienced. This limitation of the software, which as yet does not perform automatic analysis, precludes the standardization of the use of spectroscopy for all cases, making it currently feasible for use only in complex cases.

This study has some limitations, chief among which is the small sample size. Further studies involving a larger number of cases of nonadenomas are needed in order to confirm the usefulness of the proposed methodology, especially in cases of metastasis. Metastases represent a major diagnostic challenge, and an analysis of a larger number of confirmed cases with metastatic involvement will also help validate this new methodology. It is important to remember that metastatic disease without a known primary malignancy is uncommon, occurring in only approximately 4% of patients with incidentally detected adrenal masses and in less than 1% of the general population^(26-28)^. However, it seems relevant to point out that 8 (8.2%) of our 97 patients were found to have adrenal carcinoma, which is a significant proportion given the very low prevalence of this type of tumor in the general population^(5,29)^ and is of great clinical interest due to the high mortality rate^(6)^. Another limitation of our study is that our sample of nodules submitted to spectroscopy did not include any cases of collision lesions, such as coexisting adenoma and metastasis, or myelolipoma. Adrenal myelolipomas generally do not pose a diagnostic challenge, because they typically feature macroscopically visible fat on CT or MRI scans. We believe that the use of the proposed methodology for a typical lesion is questionable. Collision lesions are extremely rare and occur at an unknown prevalence^(30)^, the most common combination being that of adrenal adenoma and myelolipoma.

We hope that the use of spectroscopy at other research centers will help improve the technique. The analysis of adrenal spectroscopy is still a nascent, evolving technique that has yet to be standardized like that of spectroscopy of the prostate.

In the present study, chemical shift MRI and MRS spectroscopy demonstrated high accuracy in the differentiation between adenomas and nonadenomas. However, the fact that it was not possible to use spectroscopy in a large number of cases precludes the inclusion of the method in the clinical routine. Nevertheless, all proposed methods of measuring the SII (SII-minimum, SII-mean, and SII-maximum) presented significant and concordant results in the differentiation between adenomas and nonadenomas in our sample. Despite the technical difficulties in our case series, we found that the Lac/Cr metabolite ratio had higher accuracy than did the SII.
